# Concomitant surgery for aortic valve and lung cancer patients in an elder

**DOI:** 10.1186/s13019-020-01277-x

**Published:** 2020-09-16

**Authors:** Hongfei Xu, Tingting Tao, Liang Ma, Weidong Li, Yiming Ni

**Affiliations:** Department of Cardiovascular Surgery, The First Affiliated Hospital of Zhejiang University, Number 79 Qingchun Road, Hangzhou, China

**Keywords:** Transcatheter aortic valve implantation; lung cancer; concomitant surgery

## Abstract

**Background:**

The treatment strategy for aortic valve and lung cancer patients includes concomitant or two-stage procedures. Conventional simultaneous operations are usually performed under the median sternotomy.

**Case presentation:**

A 72-year-old man was admitted to our hospital after experiencing chest tightness after activity for two months. Aortic valve regurgitation had been confirmed when squamous cell carcinoma of the lung was discovered. The therapeutic strategy for these patients is controversial. Considering the potential risk of tumour metastasis and the risk of cardiopulmonary bypass (CPB), we recommended concomitant transcatheter aortic valve implantation (TAVI) and a lobectomy. A trans-apical TAVI with left-sided intercostal thoracotomy was successfully performed, followed by an immediate video-assisted thoracoscopic surgery (VATS) lobectomy and selective lymph node dissection.

**Conclusions:**

We suggest that a one-stage surgery of pulmonary resection following TAVI is an acceptable and safe choice after careful evaluation and should be performed as soon as possible in response to lung cancer in elderly patients with aortic valve disease.

## Background

Lung cancer can be incidentally discovered during preoperative evaluation of aortic valve regurgitation. The treatment strategy includes concomitant or two-stage surgery. The conventional simultaneous operation for valve replacement and lung resection is usually performed under the median sternotomy. Here, we describe a way to treat both diseases through concomitant trans-apical TAVI and VATS lobectomy and selective lymph node dissection, avoiding the adverse effects of CPB, repeated anaesthesia and pain without delaying lung cancer treatment.

## Case Presentation

**Fig. 1:**
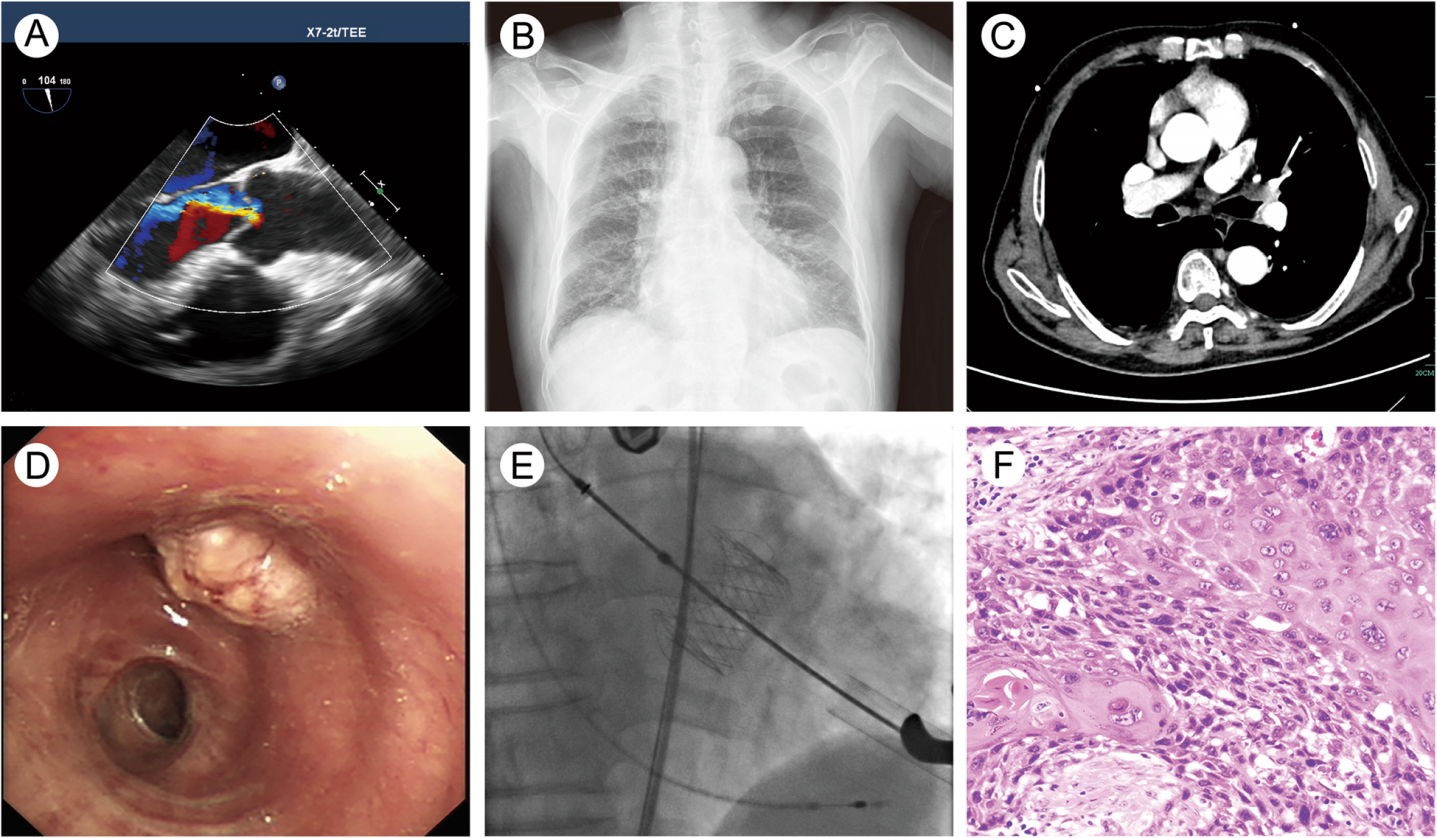
**a** Transesophageal echocardiography showed that the aortic valve was trilobal, with poor valve closure and moderate to severe regurgitation (Vmax 4.3 m/s, LVDD 62 mm, LVEF 55%). **b** The chest X-ray indicated chronic bronchitis, emphysema and two lung infections. (C) The CT scan showed a partial blockage in the left upper lobe bronchus. **d** The bronchoscope showed a tumour in the left upper lobe. The distance from the carina is more than 2 cm, and a later biopsy revealed squamous cell carcinoma. **e** The intraoperative angiography showed a 29 J-Valve was implanted from the apex of the heart. **f** The pathological findings revealed central highly differentiated squamous cell carcinoma (HE × 400)

A 72-year-old male patient was admitted to our hospital. He had been experiencing chest tightness and blood in his sputum for two months. A transoesophageal ultrasound revealed moderate to severe aortic valve regurgitation (Fig. 1a), a result of cusp prolapse due to generalized disease, and a chest X-ray indicated lung infections (Fig. 1b). Computed tomography (CT) scans before TAVI revealed a partial blockage in the left upper lobe bronchus (Fig. 1c), and a bronchoscopic biopsy revealed left upper lobe squamous cell carcinoma (Fig. 1d). In this complex situation of senile aortic valvular disease with lung cancer, we used a concomitant trans-apical TAVI with left-sided intercostal thoracotomy and then sleeve resection of the left upper lobe and lymph node dissection by right lateral decubitus. Our apical TAVI incision was located in the left fifth intercostal space and was about 3 cm in size. The patient’s lung cancer was located on the left side, so when we finished the TAVI, we changed the patient’s position from the supine position to the right lateral decubitus position. Then we disinfected and spread the towel, used the same incision of the left fifth intercostal space as an auxiliary operation hole and added a 5 cm incision in the left third intercostal space as the main operation hole. We also added a 1 cm observation hole between the seventh intercostal space to complete the sleeve resection under thoracoscopy. The operation lasted about five hours. A 29 J-Valve (JieCheng Medical Technologies, Suzhou, China) (Fig. 1e) was implanted, and the left upper lung lobe was excised through sleeve resection with negative margins. The tumour was 1.8 × 1.5 × 0.8 cm in size, and the bronchial wall was involved. Pathological findings revealed central highly differentiated squamous cell carcinoma (Fig. 1f) with the pathological stage of T2N0M0 stage IB. The patient recovered well after the operation. The drainage tube was removed on the fifth day, and the patient was discharged on the eleventh day. After the operation, he received radiotherapy for the left hilum and the left lung (Dt: 5000 cGy/25f, 200 cGy/f, 5f/W) 18 times. The aortic valve is normally functional with a mean gradient of 10 mmHg at the 12-month follow-up appointment. And the lung tumour recurred.

## Discussion and Conclusions

Lung cancer and severe valvular heart disease occasionally coexist. The therapeutic strategy for these patients is controversial. The incidental finding rate of lung nodules on CT scans for TAVI is about 18% [1]. There are no guidelines marking clear treatment recommendations in this situation. Undergoing cardiac surgery with CPB may cause dissemination of the coexisting lung cancer [2], and a four- to six-weeks delay could lead to unreliability or metastatic spreading. Conversely, undergoing lung surgery first will significantly increase the risks associated with anaesthesia and the risk of death [3]. The conventional simultaneous operation for valve replacement and lung resection is usually performed under the median sternotomy. Usually doctors will do valve surgery under CPB and then wait for protamine to antagonize heparin before performing pulmonary resection from the median incision. In addition, we know that minimally invasive valve surgery can be performed for valve diseases under CPB through a small right intercostal space incision. When the pulmonary nodule is located on the right lung, we can use the same incision on the right side to complete the lung surgery after the protamine injection. However, performing concomitant or two-stage procedures does not avoid CPB complications, including bleeding or tumour metastasis. Thus, some surgeons report that patients with both diseases can be treated with a two-stage surgery of TAVI followed by lobectomy [4]. In the past decade, TAVI has become a safe alternative strategy for treating aortic valve disease [5]. Kelpis suggested that TAVI with simultaneous pulmonary resection has a good long-term result [6]. Therefore, for this patient, we believed that a trans-apical TAVI operation and a VATS lung cancer radical operation could be completed at the same time through one left intercostal space incision, avoiding the potential for tumor metastasis afforded by CPB and the time interval before lung surgery, the risk of injury due to repeated anaesthesia and the high risk of an open-heart operation.

The number of elderly patients with simultaneous aortic valve disease and lung tumours is increasing. We suggest that a one-stage surgery of pulmonary resection following TAVI is an acceptable and safe choice after careful evaluation and should be performed as soon as possible for elderly patients with lung cancer and aortic valve disease.

## Data Availability

The datasets used and analyzed during the current study are available from the corresponding author on reasonable request.

## Abbreviations

CPB: Cardiopulmonary bypass
TAVI: Transcatheter aortic valve implantation
VATS: Video-assisted thoracoscopic Surgery
LVDd: Left ventricular diastolic diameter
CT: Computer tomography
